# Relationship of promising methods in the detection of anthracycline-induced cardiotoxicity in breast cancer patients

**DOI:** 10.1007/s00280-015-2874-9

**Published:** 2015-09-23

**Authors:** Ben F. Bulten, Hein J. Verberne, Louise Bellersen, Wim J. G. Oyen, Aida Sabaté-Llobera, Annelies M. C. Mavinkurve-Groothuis, Livia Kapusta, Hanneke W. M. van Laarhoven, Lioe-Fee de Geus-Oei

**Affiliations:** MIRA Institute for Biomedical Technology and Technical Medicine, University of Twente, Zuidhorst Room 2.65, Drienerlolaan 5, PO Box 217, 7500 AE Enschede, The Netherlands; Department of Radiology and Nuclear Medicine, Radboudumc, Geert Grooteplein 10, Nijmegen, The Netherlands; Department of Nuclear Medicine, Academic Medical Center, Meibergdreef 9, Amsterdam, The Netherlands; Department of Cardiology, Radboudumc, Geert Grooteplein 10, Nijmegen, The Netherlands; Department of Nuclear Medicine, Hospital Universitari de Bellvitge, Feixa Larga, Barcelona, Spain; Princess Maxima Center for Pediatric Oncology, Lundlaan 6, Utrecht, The Netherlands; Department of Children’s Heart Center, Radboudumc, Geert Grooteplein 10, Nijmegen, The Netherlands; Pediatric Cardiology Unit, Tel Aviv Sourasky Medical Center, Weizmann Street 10, Tel Aviv, Israel; Department of Medical Oncology, Academic Medical Center, Meibergdreef 9, Amsterdam, The Netherlands; Department of Medical Oncology, Radboudumc, Geert Grooteplein 10, Nijmegen, The Netherlands; Section of Nuclear Medicine, Department of Radiology, Leiden University Medical Center, Albinusdreef 2, Leiden, The Netherlands

**Keywords:** Breast cancer, Anthracyclines, Cardiotoxicity, 2D strain imaging, ^123^I-*m*IBG scintigraphy, Biomarkers

## Abstract

**Purpose:**

It remains challenging to identify patients at risk of anthracycline-induced cardiotoxicity. To better understand the different risk-stratifying approaches, we evaluated ^123^I-*meta*iodobenzylguanidine (^123^I-*m*IBG) scintigraphy and its interrelationship with conventional echocardiography, 2D strain imaging and several biomarkers.

**Methods:**

We performed ^123^I-*m*IBG scintigraphy, conventional and strain echocardiography and biomarker (NT-proBNP, TNF-α, galectin-3, IL-6, troponin I, ST-2 and sFlt-1) assessment in 59 breast cancer survivors 1 year after anthracycline treatment. Interobserver and intermethod variability was calculated on planar and SPECT ^123^I-*m*IBG scintigraphy, using the heart/mediastinum (H/M) ratio and washout (WO). Pearson’s *r* and multivariate analyses were performed to identify correlations and independent predictors of ^123^I-*m*IBG scintigraphy results.

**Results:**

Delayed planar anterior whole-heart ROI (WH) H/M ratios and WO were the most robust ^123^I-*m*IBG parameters. Significant correlations were observed between ^123^I-*m*IBG parameters and several conventional echo parameters, global longitudinal and radial strain (GLS and GRS) and galectin-3. The highest Pearson’s *r* was observed between delayed H/M ratio and GRS (Pearson’s *r* 0.36, *p* = 0.01). Multivariate analysis showed that GRS was the only independent predictor of the delayed WH H/M ratio (*p* = 0.023).

**Conclusion:**

The delayed planar H/M ratio is the most robust ^123^I-*m*IBG parameter. It correlates with several conventional echocardiographic parameters, GLS, GRS and galectin-3. Of these, only GRS predicts the H/M ratio.

## Introduction

Anthracyclines are widely used for (neo)adjuvant treatment of breast cancer [11, 27]. This class of drugs is associated with cardiotoxicity [3]. Anthracycline-induced cardiotoxicity (AIC) can be acute, which may lead to chemotherapeutic dose reduction, but is generally reversible. However, development of chronic AIC (i.e. ≥1 year after therapy) is often irreversible and may have a significant impact on the overall prognosis and survival of breast cancer survivors [37]. As treatment with anthracyclines resulted in significantly improved breast cancer survival over the past decades, the importance of (early) detection and prevention of potential side effects has increased. Most commonly, the possible deleterious effects of anthracyclines on left ventricular function are monitored by left ventricle ejection fraction (LVEF) measurement using multigated radionuclide angiography (MUGA) or (2D non-contrast) echocardiography [3, 17]. However, the reproducibility of echocardiography parameters varies and both techniques only detect LVEF changes that occur after considerable damage has been acquired [19, 36]. An adequate technique to identify patients at risk of cardiotoxicity (i.e. before the damage occurs) is still lacking [17].

The pathophysiology of AIC is complex and not yet fully understood. Recent research has focused on the topoisomerase-IIβ enzyme as the core defect mechanism, which is believed to induce cell death upon the formation of a complex with the anthracycline doxorubicin [24, 38]. This topoisomerase-IIβ–doxorubicin complex-induced cell death subsequently triggers a cascade of cytokine release and compensatory mechanisms, which are potential targets for early detection of AIC [7]. When myocytes die, cardiac output declines, with the release of norepinephrine (NE) in the synapse by the sympathetic nervous system as one of the first neurohumoral responses [1]. The release of NE in combination with a decreased presynaptic NE reuptake (i.e. NE transporter downregulation) leads to an increased concentration of NE in the synaptic cleft [12, 15]. *Meta*-iodobenzylguanidine (*m*IBG) is an analogue of the sympathetic neurotransmitter NE. Labelling of *m*IBG with ^123^I allows for scintigraphic assessment of sympathetic activity and may provide a measure for early detection of AIC [15]. The most commonly used methods to quantify myocardial ^123^I-*m*IBG uptake are the measurement of the heart/mediastinum (H/M) ratio and washout (WO). Increased sympathetic cardiac activity is characterized by a decreased H/M ratio and an increased myocardial washout of ^123^I-*m*IBG.

Other novel methods that may detect AIC in an early stage include 2D strain (rate) imaging with echocardiography and blood biomarkers. 2D strain (rate) imaging measures the relative deformation (i.e. stretch) of cardiac tissue in three different axes [25]. Since strain can differentiate active from passive movement, subtle regional differences can be detected long before LVEF deteriorates [31]. Blood biomarkers can be measured to provide information on cardiac pathological processes, as summarized in the cytokine hypothesis by Braunwald et al. [7]. Myocyte injury, whether it is due to hemodynamic or ischaemic stress, induces release of the compensatory prohormone N-terminal probrain natriuretic peptide (NT-proBNP), the myofibrillar protein troponin I and different cytokines including tumour necrosis factor alpha (TNF-α) and interleukin-6 (IL-6). Subsequently, activated monocytes secrete the interleukin-1 receptor family member ST2, while macrophages produce galectin-3 [7]. Furthermore, the angiogenesis promoter-soluble Fms-like tyrosine kinase receptor 1 (sFlt-1) is activated [21].

As ^123^I-*m*IBG scintigraphy, 2D strain (rate) imaging and blood biomarkers reflect different aspects of the same pathophysiological mechanism, they most likely show some interrelationship. However, knowledge on this possible correlation is still lacking. Therefore, the aim of the current study was to study the relation between ^123^I-*m*IBG scintigraphy, echocardiographic (strain) imaging and a selection of the most promising biomarkers. We aimed to study this in a homogenous group of breast cancer survivors 1 year after a potentially cardiotoxic chemotherapeutic regimen, containing anthracyclines.

## Materials and methods

### Patient selection

All adult female patients presented between October 2010 and May 2012 with breast cancer and at least 1 year after completion of (neo)adjuvant treatment with docetaxel, doxorubicin (i.e. anthracycline) and cyclophosphamide (TAC) were asked to participate in the study. Exclusion criteria consisted of major heart disease (i.e. myocardial infarction, percutaneous coronary intervention or coronary artery bypass graft) at the time of breast cancer diagnosis, renal failure at the time of cardiac evaluation, evidence of breast cancer recurrence or metastatic disease, pregnancy or breast feeding, participation in a research protocol with ionizing radiation 1 year prior to inclusion, diabetes mellitus, Parkinson’s disease or an ^123^I-*m*IBG accumulating tumour.

A detailed medical history and physical examination were obtained in all patients, with special attention to risk factors and signs and symptoms of cardiac disease. Current medication use was noted. A standard 12-lead electrocardiogram was performed and analysed for signs of cardiac disease and rhythm disturbances. The study was approved by the medical ethics committee of the Radboud University Medical Center (Nijmegen, The Netherlands), and informed consent was obtained from all patients.

### ^123^I-mIBG scintigraphy

Patient medication interfering with ^123^I-*m*IBG uptake was interrupted for at least 5 half-lives after consultation of the attending physician. Thyroid ^123^I uptake was blocked by oral administration of 400 mg potassium perchlorate 1 h before intravenous injection of 185 MBq^123^I-*m*IBG (AdreView, GE Healthcare). Patients rested for 30 min prior to injection.

Planar ^123^I-*m*IBG images were acquired in anterior and posterior view 15 min (‘early’) and 4 h (‘delayed’) after injection. Imaging was performed during 10 min using a 20 % energy window centred on the 159-keV photopeak of ^123^I, and acquired with a medium energy collimator and stored in a 128 × 128 matrix. No scatter correction was applied. Subsequently, a single-photon emission computed tomography (SPECT) of the thorax was performed 35 and 260 min post-injection, obtaining 32 frames of 60 s/frame, on a dual-head detector system, using a rotation of 180°.

### Measurements on planar images

**Fig. 1:**
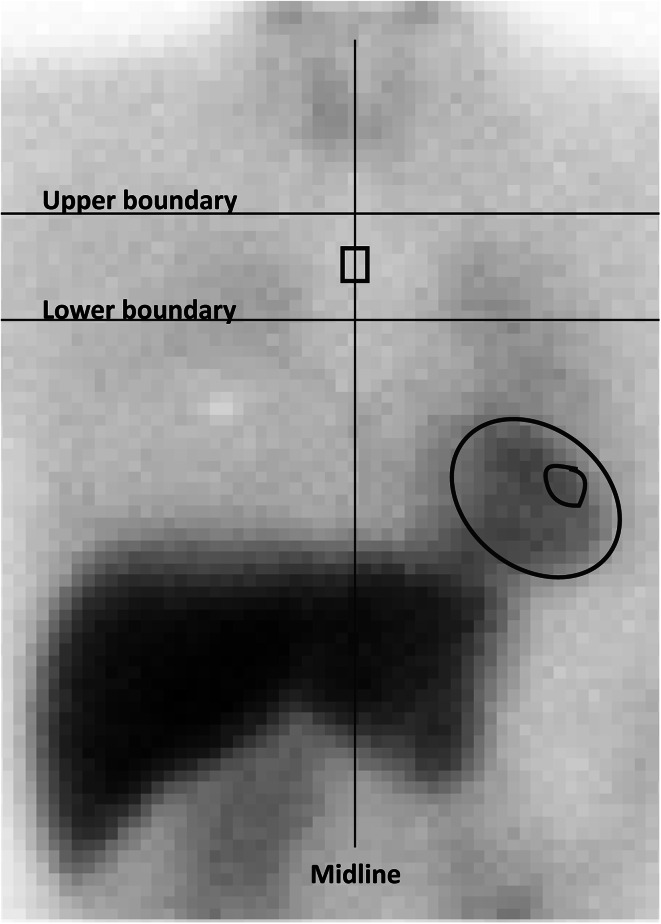
Standardized approach for the placement of the mediastinal and heart ROIs for H/M ratio determination, adapted from Somsen and Flotats [16, 30] Notice the *upper* and *lower boundary* defining the upper mediastinum and the mediastinal midline. The heart ROI consists of either a circular ROI including the left ventricle and the cavum (whole-heart ROI—WH) or a *small circular* ROI on the left ventricle lateral wall (small LV ROI; Sm)

On the anterior planar images, 10–20 pixel regions of interest (ROI) were drawn over the upper mediastinum by two observers (BB and ASL) (Fig. 1) [16, 30]. This ROI was then placed over the LV anterior wall to obtain the small left ventricular (Sm) ROI. Furthermore, a whole-heart (WH) ROI was manually determined. Both ROIs were mirrored on the posterior planar images. For all planar images, H/M ratios were calculated by dividing the cardiac average counts per pixel by the mediastinal average counts per pixel. Furthermore, a geometric mean of the heart and mediastinum counts was calculated (by means of the formula $\sqrt {{\text{countsanterior}} \times {\text{countsposterior}}}$), resulting in the geo H/M ratio. Eventually, this resulted in two measurements of the H/M ratios (i.e. anterior and geometric mean) on two time points (i.e. early and delayed) and with two ROI methods (i.e. WH and Sm).

The (background corrected) myocardial WO was defined as described by Veltman [35].

This was done for both the anterior images and the geometric mean method, using both the WH and Sm ROI delineation method. WO is expressed as a percentage.

### Single-photon emission computed tomography (SPECT) images

For SPECT image interpretation, tomographic slices were reconstructed in short axis, horizontal axis and vertical long axis planes. Early and delayed images were identically aligned so that simultaneous analysis of the image planes was allowed. Mediastinal, WH and LV (i.e. including or excluding the cavum) voxels of interest (VOIs) were visually drawn using Inveon Research Workplace 4.1 (IRW, Siemens Molecular Imaging). The mediastinal VOI had a fixed spherical shape of 20 voxels, and care was taken to exclude thyroid tissue. H/M ratios and WO were calculated with mean voxel count as described above.

### Echocardiography and biomarkers

Methods on the measurement of conventional and strain echocardiography parameters and biomarkers have been described extensively elsewhere [9, 34]. The current study focused on the interrelationship of these parameters with ^123^I-*m*IBG values. The parameters on conventional echocardiography that were studied included the internal dimensions of the left ventricle at end-diastole (LVIDd) and end-systole (LVIDs), the posterior and septal wall thickness at end-diastole (LVPWd, IVSd), left ventricular mass (LVM), left ventricular volume at end-diastole (LVEDV) and end-systole (LVESV), left atrial end-diastolic volume (LAEDV), left ventricular ejection fraction (LVEF), early (E) and late (A) diastolic transmitral peak flow velocity (E/A ratio), the systolic to diastolic pulmonary vein peak flow velocity (PV S/D ratio) and early diastolic transmitral peak flow velocity (E) to early diastolic annular velocity (e′) ratio (E/e′ ratio). Measurements of LVEDV, LVESV, LVM and LAEDV were indexed by body surface area (BSA).

The biomarkers we studied were NT-proBNP, TNF-α, galectin-3, IL-6, troponin I, ST-2 and sFlt-1. Biochemical risk factors for cardiovascular disease (cholesterol, triglycerides, HDL, LDL, glucose, HbA1C) were also determined.

### Statistical analysis

Patient age and time after treatment are expressed in years or months with range. Other patient characteristics are expressed in numbers of total and percentage. ^123^I-*m*IBG and echocardiographic values are expressed as mean ± SD. Biochemical values are expressed as median with interquartile range.

Lin’s concordance correlation coefficients (LCCs) with 95 % confidence intervals are calculated for interobserver variability and depict the correlation of measurements of two different observers. For clinically relevant agreement, the following criteria are used: LCC values <0.90, 0.90–0.95, 0.95–0.99 and >0.99 were considered to indicate poor, moderate, substantial and almost perfect agreement, respectively [26]. The coefficient of variation (CV) is the relative ratio of SD to mean and expressed in percentage. 95 % limits of agreement in Bland–Altman plots are defined as mean ± 2SD, which is numerically expressed as the coefficient of repeatability (CR; calculated as 1.96 × SD).

Correlations of the various studied methods are expressed in Pearson’s *r*. Correlations with a one-tail *p* < 0.1 were included in multivariate regression analysis, which was performed in a forward stepwise fashion. Significance was set at *p* < 0.05. All statistical analyses were performed with SPSS for Windows, version 20.0.

## Results

### Patient characteristics

Fifty-nine breast cancer survivors were included in the study. All patients had received a full dose of anthracyclines (300 mg/m^2^), except one who received 250 mg/m^2^. None of the patients had a history of major cardiac events (i.e. myocardial infarction, percutaneous coronary intervention or coronary artery bypass graft), nor did any of them indicate chest pain or express heart failure signs/symptoms. One patient presented with a known left bundle branch block. Other patient characteristics, including risk factors and medication use, are displayed in Table 1. One year after treatment the mean LVEF was 62.6 (±7). LVEF was <55 % in three patients, respectively, 35, 53 and 54 %. The observed LVEF of 35 % was due to aortic sclerosis, which was observed on conventional echocardiography and returned to normal after surgical intervention. The other two patients did not receive follow-up, since they did not meet the criteria for subclinical cardiotoxicity. None of the patients used dexrazoxane.

**Table 1:** Patient characteristics of the study group (*N* = 59)

	*N* (%)	Median (range)
*Clinical characteristics*		
Age in years		52 (36–69)
Time after treatment in months		12.5 (10–14)
*Risk factors*		
Smoking	13 (22)	
Hypertension^a^	18 (30)	
Family history^b^	9 (16)	
HbA1c >53 mmol/L	1 (2)	
BMI >30 kg/m^2^	13 (22)	
*Medication use*		
ACE/ATII	2 (3)	
Beta blocker	6 (10)	
Diuretic	9 (15)	
Statin	5 (9)	
Calcium antagonist	2 (3)	
*Treatment characteristics*		
Tumour side		
Left	28 (46)	
Right	24 (39)	
Both	11 (12)	
Radiation left thorax side	22 (37)	
Total radiation in gray		65 (45–142)
Cumulative anthracycline dose in mg/m^2^		300 (250–300)

^a^Systolic blood pressure >140 mmHg and/or use of antihypertensive medication

^b^Presence of coronary artery disease in a first-degree family member at <55 years in men or <65 years in women

### Planar H/M ratio and WO

Anterior and geometric mean H/M ratios and WO were obtained in all patients on both early and delayed ^123^I-*m*IBG images and by both observers. All anterior H/M ratios and WO were significantly lower than geometric mean H/M ratios and WO (Table 2).

**Table 2:** Planar H/M ratio and WO characteristics

	Anterior			Geometric mean			Intermethod ∆	
	Mean		LCC	Mean		LCC	Mean	
	SD	CV (%)	95 % CI	SD	CV (%)	95 % CI	SD	CR
*WH*								
Early H/M ratio	2.71		0.90	2.99		0.91	0.28^†^	
	0.44	16.2	0.83–0.94	0.40	13.4	0.85–0.94	0.21	0.41
Delayed H/M ratio	2.72		0.92	2.82		0.92	0.10^†^	
	0.51	18.8	0.86–0.95	0.43	15.2	0.87–0.95	0.21	0.41
WO (%)	21.9		0.83	27.5		0.80	5.6^†^	
	13.1	59.8	0.74–0.89	10.2	37.1	0.69–0.87	5.6	11.0
*Sm*								
Early H/M ratio	2.80		0.69	3.05		0.49	0.25^†^	
	0.53	18.9	0.56–0.78	0.46	15.1	0.34–0.62	0.24	0.48
Delayed H/M ratio	2.83		0.71	2.92		0.57	0.09^†^	
	0.56	19.8	0.58–0.80	0.47	16.1	0.40–0.70	0.24	0.47
WO (%)	20.0		0.53	26.0		0.57	6.0^†^	
	14.0	70.0	0.36–0.67	10.7	41.1	0.37–0.71	7.9	15.5

*H/M ratio* heart/mediastinum ratio, *WO* washout, *WH* whole-heart ROI, *Sm* small left ventricle ROI; *SD* standard deviation, *CV* coefficient of variation (SD/mean), *LCC* Lin’s correlation coefficient, *CI* confidence interval, *CR* coefficient of repeatability (1.96 × SD), Δ = difference

^†^ *p* < 0.001

#### Interobserver and intermethod variability

**Fig. 2:**
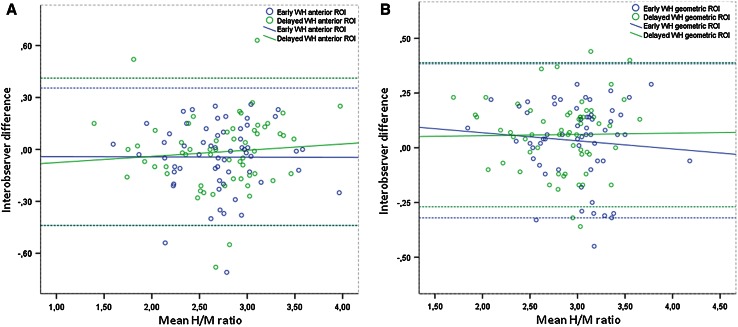
Bland–Altman plots of interobserver difference versus mean of planar anterior (**a**) and geometric (**b**) WH H/M ratio, both on early and on delayed images. *Butted lines* represent 95 % limits of agreement. A. Mean differences: early images −0.04 (95 % CI −0.10 to 0.01), *R* ^2^ = 1.0 e^−5^; delayed images −0.01 (95 % CI −0.07 to 0.04), *R* ^2^ = 0.01. B. Mean differences: early images 0.03 (95 % CI −0.01 to 0.08) *R* ^2^ = 0.01; delayed images 0.06 (95 % CI 0.02–0.10), *R* ^2^ = 2.2 e^−4^

**Fig. 3:**
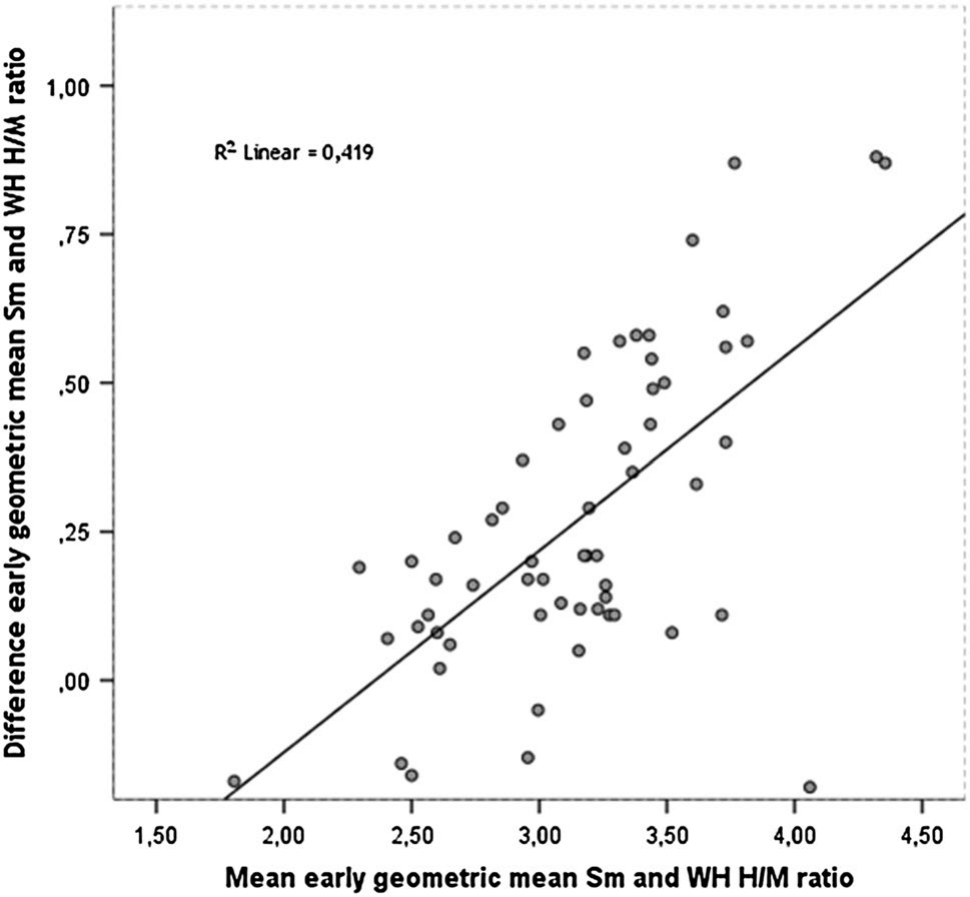
The dilution effect. Early geo WH versus Sm H/M ratio showed an LCC of 0.87 (95 % CI 0.81–0.91), *R* ^2^ = 0.42. The increasing intermethod difference is due to the dilution effect: the difference between cavum and myocardium increases when the myocardium has high ^123^I-*m*IBG uptake (i.e. the normal heart), and the ROI inclusion of blood pool (i.e. WH ROI) will account more heavily to the average heart count. Other LCCs were 0.79 (95 % CI 0.71–0.86, early anterior), 0.87 (95 % CI 0.81–0.91, delayed anterior) and 0.82 (95 % CI 0.74–0.88, delayed geo). Intermethod analysis of WO showed a poor correlation of 0.82 (95 % CI 0.72–0.88) for anterior images and a moderate correlation of 0.91 (95 % CI 0.85–0.94) for delayed images

Interobserver correlations of WH H/M ratios were moderate, of Sm H/M ratios and WO poor (Table 2). However, mean interobserver differences were small for WH H/M ratio (Fig. 2). Intermethod variability, describing the correlation of WH and Sm ROI definition by one observer, demonstrated poor LCCs (Fig. 3).

### SPECT H/M ratio and WO

IRW-derived H/M ratios are summarized in Table 3. Early H/M ratios could be calculated in 52 patients (88 %), delayed H/M ratios in 43 patients (73 %). This discrepancy was mainly caused by technical or acquisition protocol difficulties (e.g. hardware failure, data file corruption).

**Table 3:** IRW-derived ^123^I-*m*IBG SPECT indices

	Mean (SD)	
	WH	LV
Early H/M ratio	4.28 (0.96)	4.39 (0.99)^†^
Delayed H/M ratio	4.35 (1.24)	4.45 (1.27)^†^
Washout rate (%)	20.9 (14.9)	21.5 (14.4)

*WH* whole-heart ROI, *LV* left ventricle ROI

^†^ *p* < 0.001

#### Intermethod variability

**Fig. 4:**
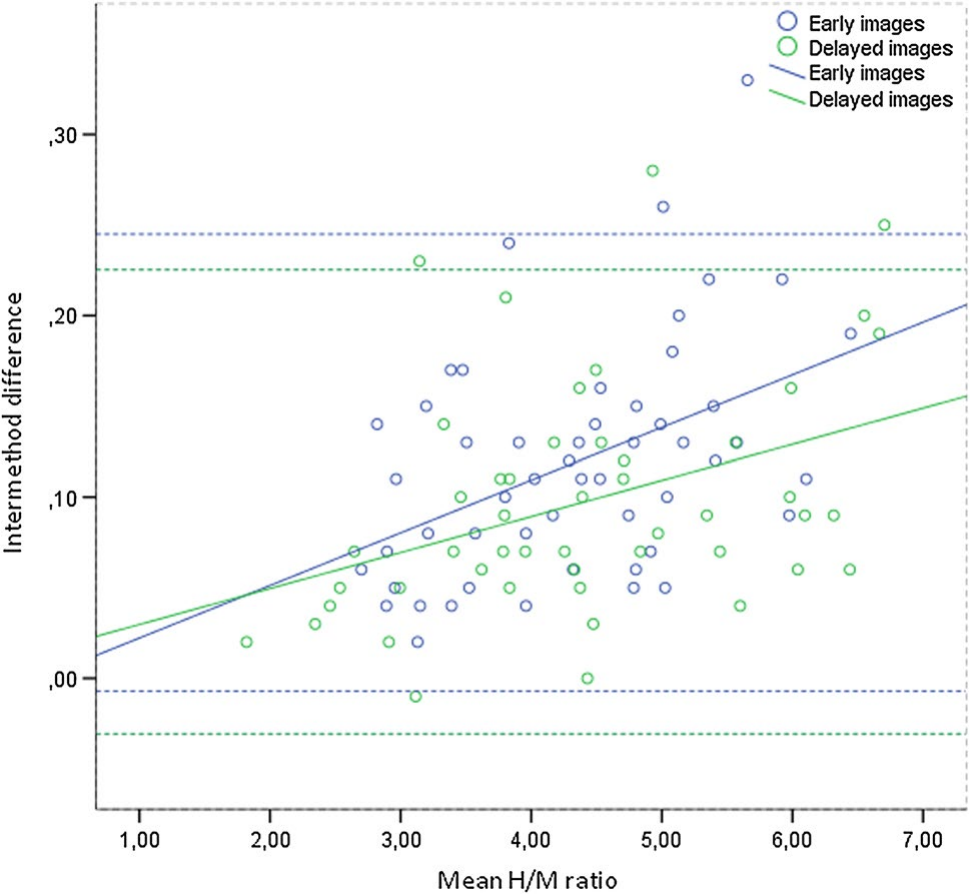
IRW-derived ^123^I-*m*IBG SPECT intermethod differences. Early WH versus LV H/M ratio: mean difference 0.12, LCC 0.99 (95 % CI 0.985–0.994), *R* ^2^ = 0.20. Delayed WH versus LV H/M ratio: mean difference 0.10, LCC 0.996 (95 % CI 0.993–0.997), *R* ^2^ = 0.15. Butted lines represent 95 % limits of agreement. The differences can be accounted for by the dilution effect. WO mean difference −0.5 %, LCC 0.993 (95 % CI 0.987–0.996), not included in figure

The intermethod correlation of SPECT-derived WH versus LV H/M ratio (both early and late) and WO was almost perfect (LCC 0.99). Mean differences were very small (Fig. 4).

### SPECT versus planar

The WH method is the only method used for both planar and SPECT images. Therefore, correlation between SPECT and planar H/M ratio was calculated specifically for that method. We observed a Pearson’s *r* of 0.33 (*p* < 0.05) for the early images, *r* = 0.35 (*p* < 0.05) for the late images and *r* = 0.76 (*p* < 0.001) for WO.

### Conventional and strain (rate) echocardiography and biomarker results

Although additional data on conventional echocardiographic and strain (rate) imaging and biomarkers are described in earlier work, [9, 34] for the purpose of completeness, an overview of the obtained results is presented in Table 4.

**Table 4:** Conventional and strain (rate) echocardiography and biomarker results

	Study population		Reference value (RV)	Abnormal values^a^	
	*N*	Mean (SD*)*		<RV	>RV
*Conventional echocardiography*					
EF (%)	59	62.6 (7.1)	≥55	4 (7)	N/A
LVEDV/BSA (ml/m^2^)	58	46.2 (10.0)	35–75	7 (12)	1 (2)
LVESV/BSA (ml/m^2^)	58	18.0 (6.3)	12–30	9 (16)	1 (2)
LVIDd (cm)	59	4.6 (0.5)	3.9–5.3	3 (5)	7 (12)
IVSd (cm)	59	0.9 (0.1)	0.6–0.9	0	14 (24)
LVPWd (cm)	59	0.9 (0.1)	0.6–0.9	0	15 (25)
LVM/BSA (g/m^2^)	59	77 (17)	44–88	0	12 (20)
LAEDV/BSA (ml/m^2^)^a^	57	22 (6)	22 (±6)	1 (2)	2 (4)
E/e′ ratio	52	6.5 (2.3)	<8	N/A	10 (19)
E/A ratio	58	1.2 (0.3)	1.3 (±0.3)	3 (6)	3 (6)
PV S/D ratio	54	1.4 (0.4)	1.2 (±0.2)	3 (6)	15 (28)
*2D strain (rate) echocardiography*					
GLS	57	−17.7 (3.1)	−17.8 (2.1)	2 (4)	6 (11)
GLSR	57	−0.87 (0.2)	−0.87 (0.1)	3 (5)	2 (4)
GRS	42	38.0 (10.0)	40.5 (11.4)	1 (2)	0
GRSR	41	1.45 (0.4)	2.20 (0.6)	0	0
GCS	42	−18.9 (4.6)	−20.3 (2.6)	3 (7)	7 (17)
GCSR	41	−1.03 (0.2)	−1.72 (0.3)	0	24 (57)
*Median (interquartile range)*					
Biomarkers					
NT-proBNP (pg/ml)	59	119 (127)	18–50 years: <170	N/A	10 (17)
			50–60 years: <250		
			60–70 years: <300^†^		
Troponin I (µg/l)	59	<0.2 (<0.2)	<0.2^†^	N/A	0 (0)
TNF-α (pg/ml)	55	<2.8 (1.5)	<10^‡^	N/A	4 (7)
Galectin-3 (ng/ml)	55	12.9 (3.6)	<17.6^‡^	N/A	4 (7)
IL-6 (pg/ml)	55	<3.12 (<3.12)	<10^‡^	N/A	0 (0)
ST2 (ng/ml)	55	10.6 (14.4)	4.9–19.9^‡^	0 (0)	0 (0)
sFlt-1 (pg/ml)	55	<320 (<320)	Not detectable^‡^	N/A	0 (0)

*EF* ejection fraction, *LVEDV* left ventricular end-diastolic volume, *BSA* body surface area, *LVESV* left ventricular end-systolic volume, *LVIDd* left ventricle internal dimension at diastole, *IVSd* interventricular septum at diastole, *LVPWd* left ventricular posterior wall at diastole, *LVM* left ventricular mass, *LAEDV* left atrial end-diastolic volume, *E/e′ ratio* early diastolic transmitral peak flow velocity (E) to early diastolic annular velocity (e′) ratio, *E/A ratio* early (E) and late (A) diastolic transmitral peak flow velocity ratio, *S/D ratio* systolic/diastolic ratio, *GLS* global longitudinal strain, *GLSR* global longitudinal strain rate, *GRS* global radial strain, *GRSR* global radial strain rate, *GCS* global circumferential strain, *GCSR* global circumferential strain rate, *N/A* not applicable

^†^RV as established by the Radboudumc chemical laboratory

^‡^RV as stated in manufacturer’s protocol

^a^Abnormal value is outside 2 SD when reference is defined as a mean ± SD

### Correlation of ^123^I-mIBG with conventional and strain echocardiography and biomarkers

Significant correlations between the delayed planar WH H/M ratios and LVEDV/BSA, LVESV/BSA, IVSd, LVM/BSA, E/A ratio (conventional echocardiography), GLS, GRS (strain echocardiography) and galectin-3 (biomarker) were identified (Table 5). The most significant correlation was observed for GRS (Pearson’s *r* 0.36, *p* = 0.01), the least significant for E/A ratio (Pearson’s *r* 0.19, *p* = 0.08). LVEDV/BSA, LVESV/BSA, IVSd, LVM/BSA, GLS and galectin-3 showed an inverse correlation, while E/A ratio and GRS showed a direct correlation.

**Table 5:**
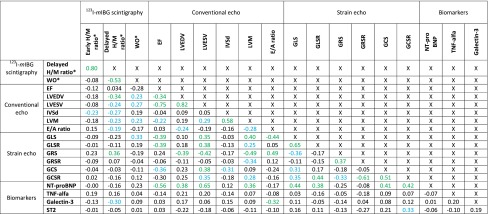
Overview of interrelationship of different parameters

* Planar anterior WH region. *Green* *p* < 0.01. *Blue* *p* < 0.05. H/M ratio = heart/mediastinum ratio; *WO* washout, *EF* ejection fraction, *GLS* global longitudinal strain, *GLSR* global longitudinal strain rate, *GRS* global radial strain, *GRSR* global radial strain rate, *GCS* global circumferential strain, *GCSR* global circumferential strain rate, *LVEDV* left ventricular end-diastolic volume, *LVESV* left ventricular end-systolic volume, *IVSd* interventricular septum at diastole, *LVM* left ventricular mass, *E/A ratio* early (E) and late (A) diastolic transmitral peak flow velocity ratio. LVEDV, LVESV and LVM are indexed by body surface area. Troponin, IL-6 and sFlt-1 have not been tested because there were no abnormal results

#### Multivariate analysis

Since delayed anterior WH H/M ratio seems to be the most robust measurement in our population, we aimed to identify by which parameters it was influenced. Multivariate analysis showed that GRS was the only independent predictor of the WH H/M ratio (standardized *β* = 0.36, *p* = 0.023).

## Discussion

In the current study we examined the interrelationship of an extensive panel of potential parameters for the early detection of chronic AIC, in a homogenous group of breast cancer survivors 1 year after treatment. There was a significant correlation between the delayed planar WH H/M ratio and several conventional echocardiographic values, GLS, GRS and galectin-3. Furthermore, GRS was identified as an independent predictor of the late planar WH H/M ratio.

Because high reproducibility is an important requirement for any diagnostic modality, we first evaluated the interobserver and intermethod variability of different methods of H/M ratio calculation to identify the most robust one, since the delineation method is a main factor hampering widespread clinical use of cardiac ^123^I-*m*IBG scintigraphy [10, 14, 35]. In most clinical studies, the WH ROI on anterior planar images is used [2, 6, 20, 33]. We showed that indices based on the geometric mean based did not improve reproducibility, nor was there a difference between early and delayed indices. Although a Sm ROI reduces a possible ‘dilution effect’ (Fig. 3), it is inferior to the WH ROI because of its high observer variability (Table 2). The Sm ROI should therefore not be used.

The addition of SPECT to ^123^I-*m*IBG scintigraphy might increase the diagnostic potential of this technique. Although most studies focus on regional sympathetic innervation rather than a global SPECT assessment, Chen et al. [13] described a high reproducibility and accuracy of global ^123^I-*m*IBG SPECT evaluation, which allows to separate heart failure patients from healthy controls. We observed a high correlation between planar and SPECT-derived parameters, especially for WO. Furthermore, SPECT H/M ratios were systematically higher than planar ratios, which is most likely caused by an overestimation of background activity on planar images [13]. Although SPECT reconstructions result in a more accurate calculation of the H/M ratio, SPECT image acquisition and reconstruction are time-consuming and performed with different protocols, yielding divergent H/M ratio ranges, thus hindering standardization [13]. Hence, global ^123^I-*m*IBG SPECT imaging, although promising, does not provide sufficient added value to be recommended for use in daily clinical practice.

Several conventional echocardiographic parameters, GLS, GRS and galectin-3 showed a correlation with WH H/M ratio, but only GRS proved to be an independent predictor.

GRS measures the relative deformation of the cardiac left ventricular wall in the radial direction (i.e. LV wall thickening), yielding a positive strain value during systole. In AIC, functioning myocytes are replaced by non-contracting fibrotic cells [32], which leads to impaired cardiac thickening and a decrease in GRS. Due to the sympathetic response to myocardial damage, the H/M ratio will also decrease. Therefore, theoretically one would expect a direct relationship between GRS and WH H/M ratio, which was confirmed by the results of our study. Since GLS measures shortening of the myocardial wall in the longitudinal axis, it is defined as a negative value and an inverse relationship with WH H/M ratio is expected. The results of our study indeed demonstrate an inverse correlation between GLS and WH H/M ratio, although not strong enough to predict the WH H/M ratio. No correlation, however, was observed between GCS and WH H/M ratio.

Of the studied biomarkers, only the novel blood biomarker galectin-3 showed a significant correlation with the WH H/M ratio. Galectin-3 is a protein expressed by macrophages and believed to be a mediator of the profibrotic pathway, stimulating cardiac fibroblasts to proliferate and deposit collagen [22]. Galectin-3 concentrations are elevated in patients with acute HF and predict an adverse outcome [29]. In a recent study by Ky et al., no association between cardiotoxicity and galectin-3 was found, although follow-up only lasted 6 months [22]. Other studies on this issue have not been performed in adults, but a recent study in paediatric patients showed an (non-significantly) increased level of galectin-3 at least 2 years after anthracycline treatment [5].

Conventional echocardiographic parameters that correlated with the WH H/M ratio included LVEDV/BSA, LVESV/BSA, IVSd, LVM/BSA and E/A ratio. The correlation of the WH H/M ratio and LVEDV, LVESV, IVSd and LVM displayed an inverse nature, which means that both cardiac volumes and cardiac wall diameters increase as the WH H/M ratio drops. This is an interesting finding, since typical AIC in adults presents as a dilated cardiomyopathy, featuring increased ventricle sizes and thin walls [18]. A possible explanation for this increased wall diameter is a compensatory myocyte hypertrophy in the dilated heart. This pattern has been described before in childhood cancer survivors [5, 23]. Another interesting finding is that the relative deformation (represented by the GRS and GLS) decreases, while the cardiac wall diameter increases. This probably reflects the replacement of active myocytes by passive (fibrotic) tissue and concurrent myocyte hypertrophy. Furthermore, we observed a (weak) direct correlation of the E/A ratio with the WH H/M ratio, indicating a concurrent decrease in the E/A ratio with the WH H/M ratio. The E velocity indicates diastolic filling, which decreases gradually in normal subjects. The A velocity reflects the active atrial contraction just before end-diastole and normally becomes more important in elderly patients, resulting in an E/A ratio approaching 1 [8]. However, a decreased E/A ratio could also imply diastolic dysfunction, indicating AIC, although opinions on the usefulness of the E/A ratio differ [4, 28].

The main limitation of the current study is the lack of baseline and follow-up measurements, so we could not assess the change in parameters over time. However, the aim of the study was not to detect a change in certain parameters, nor to predict AIC, but to assess the interrelationship of different interesting parameters in the pathophysiological process of AIC.

Although the correlations of certain parameters have been studied, for example strain (rate) imaging and troponin or conventional echocardiography and strain (rate) imaging, they have not been studied for their correlation with ^123^I-*m*IBG parameters. Furthermore, we focused on a homogenous group of breast carcinoma survivors with potential AIC damage, which is a patient group that has not yet been studied properly.

With the current study we have identified the relationship of the WH H/M ratio with 2D strain imaging, biomarkers and conventional echocardiography 1 year after anthracycline-based chemotherapy. This sheds some light on the complex pathophysiology of AIC, enabling future studies to identify appropriate parameters for the detection of AIC.

## Conclusions

Delayed planar WH H/M ratio is the most robust ^123^I-*m*IBG parameter. It is correlated with several conventional echocardiographic parameters, GLS, GRS and galectin-3. Of these, only GRS is an independent predictor of the WH H/M ratio. Future studies should concentrate on a combination of ^123^I-*m*IBG scintigraphy, MUGA, echocardiographic strain, CMR and biomarkers, preferably in a prospective multicentre trial with long-term follow-up in breast cancer survivors.
